# PATHOS: Pervasive at Home Sleep Monitoring

**DOI:** 10.1155/2008/290431

**Published:** 2008-04-17

**Authors:** Ian Obermiller, Sheikh I. Ahamed

**Affiliations:** Mathematics, Statistics and Computer Science Department, Marquette University, Milwaukee, WI 53201-1881, USA

## Abstract

Sleeping disorders affect a large percentage of the population, and many of them go undiagnosed each year because the method of diagnosis is to stay overnight at a sleep center. Because pervasive technologies have become so prevalent and affordable, sleep monitoring is no longer confined to a permanent installation, and can therefore be brought directly into the user home. We present a unique solution to the problem of home sleep monitoring that has the possibility to take the place of and expand on the data from a sleep center. PATHOS focuses not only on analyzing patterns during the night, but also on collecting data about the subject lifestyle that is relevant and important to the diagnosis of his/her sleep. PATHOS means “evoking emotion.” Here, we mean Pathos will help us to keep healthy: both mentally and physically. Our solution uses existing technology to keep down cost and is completely wireless in order to provide portability and be easily to customize. The daytime collection also utilizes existing technology and offers a wide range of input methods to suit any type of person. We also include an in-depth look at the hardware we used to implement and the software providing user interaction. Our system is not only a viable alternative to a sleep center, it also provides functions that a static, short-term solution cannot provide, allowing for a more accurate diagnosis and treatment.

## 1. INTRODUCTION

Sleep problems affect more than 70 million people in the United States alone [1]. Yet, the public as a whole is largely unaware of the causes and consequences of the disorder that one in four people have. Additionally, the majority of sleep disorders go undiagnosed and untreated. Sleep disorders range from snoring to insomnia, and can be as serious as obstructive sleep apnea, when a person stops breathing multiple times during the night. Because sleep problems cost Americans over *$*100 billion a year in lost productivity and medical expenses, it is imperative that more people are diagnosed and treated for such a common illness.

Sleeping disorders are usually diagnosed only when a person goes to a sleep center to have diagnostics taken over night. During such a study, known as a polysomnogram, a patient goes to a sleep center, spends around 45 minutes getting hooked up to a machine via electrodes, sensory belts, a microphone, and various other sensors. After the process, which is painless and made to be as nonintrusive as possible, the patient goes to sleep and the numbers are gathered. There is no doubt that this method of sleep monitoring works, or that it is the main mode of diagnosing many important sleeping disorders. However, sleep centers have several drawbacks, including cost, time, and environment.

In order for a truly universal system for monitoring sleep and diagnosing disorders to become a reality, it must be moved from the center to the home. Although this is not a new idea, the technology has only recently caught up with the proposal. There is a continual research to improve the sensors used in polysomnography. Researchers at the University of Aizu [2], for example, have created an under-pillow sensor to measure respiratory rhythm and pulse during sleep. A more complete system, with more sensors, has been designed at the University of Cairo [3] and is presented with a detailed hardware design. The University of Washington [4] has researched using inexpensive multimodal sensors to detect sleep conditions in a completely noninvasive manner.

The majority of previous research in the area of polysomnography technology has been directed toward the development hardware designs that would be more practical in a sleep center than in a home. The aim of the project at the University of Cairo [3], however, was a home monitoring solution, but its purpose was mainly to decide whether or not a person should go through with a full evaluation at a dedicated sleep center.

Our approach to the field of polysomnography combines several aspects of the previous research and seeks to provide an all-encompassing solution that negates the need for a person to ever spend the night at a costly sleep center. The three main goals of PATHOS are to be (1) simple and inexpensive, using existing technology, (2) performed in a natural environment, namely, the subject's home, and (3) a continuous solution, not limited to gathering data during the night. For night monitoring, we propose using a mesh network of simple sensors that wirelessly transmit data to a subject’s mobile device or pc. The sensors are inexpensive and require interpretation which can be performed by small motes which form the nodes of the network. Additionally, data regarding factors that affect sleep is collected throughout the day using the subject’s cell phone. Because the solution is contained entirely at the person’s home, the steep cost and unfamiliar location of a sleep center can be bypassed. Furthermore, data can be collected over a longer period of a time, producing a more accurate diagnosis.

Our paper is laid out as follows: Section 2 describes the motivation for our choice of design; Section 3 details the characteristics of our approach; Section 4 explains our approach; Section 5 focuses on our implementation; Section 6 contains information collected from a user survey and evaluation of our proposal; Section 7 discusses in greater depth related research; and finally, Section 8 presents the conclusion or our plan and the direction of our future work.

## 2. MOTIVATION

Consider the following scenarios.

### 2.1. Scenario one

Arin is an older woman who has never suspected that she had a sleeping disorder. Because an overnight sleep center is unpractical and far too expensive, she is never diagnosed for a disorder even though she is frequently tired during the day and never feels fully rested.

### 2.2. Scenario two

Grahame is a middle aged man who suspects he may have a sleeping disorder. However, he is uncomfortable staying overnight in an unfamiliar place and does not have the time due to his busy work week, so he refuses to go to a sleeping center for a diagnosis. Years later, his wife convinces him to go, and he finds that he has sleep apnea, a dangerous disorder that could have been treated long ago and has resulted in many days and nights of needless symptoms.

### 2.3. Scenario three

Finally, consider Joye, a young woman who also suspects she may have a sleeping disorder. Because there are no other options, she goes to a sleep center for diagnosis three nights in a row. During the night, she is more restless than usual. The next day, she is asked a series of questions about her lifestyle, and the doctors conclude that she has a minor sleep disorder. In reality however, she has a more major sleep disorder, and her lifestyle is making it progressively worse. The short amount of time spent monitoring her sleep and the survey that reflected her opinions about herself more than how she actually acts misrepresented her situation and caused a misdiagnosis.

### 2.4. Interpretation

Our motivation for PATHOS stems from the previous scenarios. First, a sleep center is not the most practical way for a person to be diagnosed. They are costly due to sophisticated equipment and the trained professionals who must operate the equipment. Second, not everyone has time to stay overnight somewhere multiple times a week. Third, it may be very uncomfortable for someone to sleep in a foreign place, which may lead them to sleep differently than usual because of nerves. And, if the readings at a sleep center are sufficiently different than they would otherwise be at home, a misdiagnosis could occur.

## 3. CHARACTERISTICS

The characteristics that make our approach to polysomnography novel and unique are the following.

### 3.1. Cost effective

Polysomnography is typically a very costly process, especially when done at a sleep center. Even portable systems available today are not inexpensive enough for the average person to be compelled to verify whether or not she has a sleeping disorder, especially if it is a minor one. Our system uses inexpensive, multimodal sensors and takes advantage of the existing technology a user would already have in her home, such as computer, cell phone, or PDA. In addition, the system can handle multiple users, if, for instance, a couple wanted to monitor both of their sleeping habits but only wanted to have one unit. They could trade-off each night, indicating who is wearing the sensors, and send in separate data during the day.

### 3.2. Accurate

In order to be comparable to the diagnosis a sleep center would give, a portable solution must keep accuracy as an important goal. To balance cost and accuracy, the chosen sensors are in some cases multifunctional (the pulse oximeter) and in all cases deliver the best results for the price. Also, because our approach can be used as a long-term monitoring solution, extreme sensor readings will average out over the longer period of time, producing a more accurate assessment.

### 3.3. Reliable

Because we have designed our sleep monitor with long-term monitoring as an objective, the system itself must be reliable. It should be able to be used consistently for weeks or even months at a time without degrading in accuracy or requiring any maintenance on the part of the user. Although it is impossible to prepare for all situations that may occur, the system is easily customizable for any age of the user, allowing it to respond to unique needs more effectively than a “one size fits all” solution could.

### 3.4. Privacy aware

Because there is a system that monitors sleeping habits and collects lifestyle data, it is natural that users would want to control the use of their confidential information. Our solution allows the user to customize how their information is shared and who can have access to it. By default, only the doctor who will diagnose the user will have access to the information, and data on the system itself will be restricted to the specific user who collected it.

### 3.5. Simple yet meaningful GUI

One of the major target age groups of an easy-to-use, portable sleep monitoring system is the elderly. As time progresses, the older age groups will become more and more familiar with computers and mobile devices, but to accommodate everyone now and in the future, the user interface of our application is very simple. Text is easy to read, and most importantly a new user should have little problems utilizing the full potential of the system. For the completely technophobic, it can be nearly autonomous, requiring the user to simply wear the sensors and then visit the doctor when collection is complete. However, it will also cater to the other end of the spectrum by being very customizable and revealing more options to more experienced users.

### 3.6. Easy and noninvasive to setup and use

**Figure 1:**
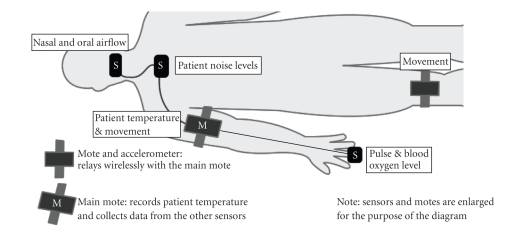
Diagram of the sensor network used for nighttime monitoring.

A user will be expected to use the system every night in order to get an accurate assessment of her sleeping patterns. For this reason, the device must be easy to setup each night and must not interfere with a good night’s rest. Pervasive technologies make this especially easy, but present some challenges as well. The communication between devices is wireless, so the user does not need to worry about many wires being tangled up during sleep. However, the number of wireless motes needs to be balanced as well, because the more parts a system contains the less easy it is to setup and use. Because of the expandability of the system, it is easy to incorporate many wireless nodes or stick to fewer nodes with more sensors attached to each one. Figure 1 shows one such configuration.

In addition to the nighttime sensor network, the daytime data collection must be quick and simple as well. By allowing the user multiple ways to enter information, she can choose the most convenient way and is therefore more likely to report throughout the day.

### 3.7. Portable

Portability is the key aspect that draws users to our system. Besides the advantages of allowing a user to sleep in her own bed each night and not be bothered by traveling to and from a sleep center; the system is also readily portable to be taken to a different sleeping location. For instance, when the user goes on vacation or temporarily is staying at another place, the system can be brought along; data collection does not have to stop.

### 3.8. Customizable

For a system to be truly universal, it must be easy to customize for different users. In our system, this is accomplished in two ways. First, as explained in Section 3.6, our wireless sensor network allows nodes to be easily added and removed. Sensors that are not applicable to a certain users diagnosis can be left out, saving time and money. Further, if a doctor believes a patient needs more advanced monitoring, additional sensors can be added to the system to provide a more detailed report. Second, the main aspects of the system can be used in several different ways to achieve the same end result. See Section 6B for an example of multiple input methods.

Such a customizable system would lead to separate base configuration for different user groups, for example, divided by age.

On the one hand, an elderly person may be technophobic, and would want a system that requires very little interaction on his part. His system could include a passive infrared video monitor that could be setup once and then used every night. Also, instead of entering data throughout the day, he could enter a simple phone survey once each night. All the data collected would be automatically sent to the doctor and analyzed without any interaction.

A middle aged person, on the other hand, would perhaps want a great deal of control over his system. He would choose the most accurate sensors that attach directly to his body, such as the accelerometer and thermistors for airflow, even though for other people they may be more difficult to setup. Although he does not use texting for cell phone data entrance, he periodically takes a quick survey from his work or home computer to enter daytime information. Every morning when he wakes up he checks out his sleeping and daytime scores, and looks to see how he can improve his sleep by changing his habits.

There is also the case of a younger person, who does not mind entering her data throughout the day via simple text messaging. At night however, she prefers a similar setup to the elderly person: easy to setup and quick to activate. Also like the elderly person, she prefers to have her doctor look at the data and thus has it sent directly to him.

The preceding customizations are only a few ways by which the system could be configured. It is adaptable to any circumstances, and can be updated with new sensors just as easily as old ones can be removed.

## 4. OUR APPROACH

We have focused on making PATHOS low cost, suitable for home use, and most importantly a continuous, long-term system. Our approach can be split into three major sections: nighttime data collection, daytime data collection, and analysis.

### 4.1. Nighttime data collection

In order to make a sensor-based system that is user friendly and not too invasive during sleep, we will use a network of sensors and motes that communicate wirelessly using the Zigbee protocol. Figure 1 details a possible setup for the patient during nighttime monitoring. Three of the sensors are connected via wires to the main mote located on the person arm. The nasal and oral airflow sensors are thermistors that measure the patient’s breathing, a characteristic especially important when diagnosing sleep apnea. There is also a microphone near the base of the subject’s neck to record sound such as snoring. The pulse and blood oxygen sensor uses a simple finger clip-on, and is then connected to the main mote by a wire running up the arm. Because of their proximity to the main mote, it would have been more cumbersome for the sensors to communicate wirelessly via their own motes. The main mote itself contains an accelerometer to measure arm movement and a thermometer to measure the subject’s temperature. The leg movement sensor (accelerometer) is connected to its own mote and transmits data to the main mote wirelessly. Additional sensors could be added wirelessly to the network very easily. For example, a simple passive infrared camera could be setup to record nighttime sleep movement patterns with more detail than the accelerometers could.

The setup that we have depicted is one of many. The system can be adapted to include more sensors in different ways, depending on the age group of the user, and what and how they want their sleep to be monitored.

Not pictured in the diagram is the receiving mote connected to the person’s personal computer. If the pc is in the same room, the mote connected to it could be used to record the ambient pressure and humidity of the sleeping environment. If not, another mote could easily be added to the network and placed on a bedside desk. The main mote on the patients arm cannot be used to measure ambient temperature and humidity because it may be covered by blankets or moved frequently during sleep.

### 4.2. Daytime data collection

The major aspect that differentiates our approach from the others is that we seek to continuously monitor the person’s lifestyle and habits that factor into the condition of her sleep. This allows us to make a more accurate assessment of factors that influence sleep and weigh the data collected during the night accordingly. Since many sleep assessments rely on survey data to collect habits and lifestyle information from patients, most of which are less than accurate, an easy to use system that gathers information as it happens could be a valuable resource for doctors diagnosing a sleep disorder. Some of the factors that will be monitored throughout the day are eating and drinking patterns (caffeine, alcohol, heavy/light meals, snacking, drinking before bed), exercise patterns (frequency, time of day, difficulty), and lifestyle (smoking, relaxing or stimulating activities before bedtime, sleeping schedule, amount of fatigue, and stress level). This data could be entered in a variety of ways, but must be convenient for the user or it will not get entered at all. Therefore, the system will rely on everyday technology that the user already has access to. For instance, she could enter information through a text message or voice automated prompt on a cell phone. Or she could go to a website and fill out a quick survey form. If none of the above is a viable option, the user could keep track in a paper log and enter the data manually later in the day.

### 4.3. Data analysis

All of the data collected throughout the day and night is relayed to a central location, which could be on the user’s pc or at a central database accessed through the internet. The central database would allow access to the patient and valid doctor’s only to insure user privacy. Because data is stored on a computer or server, there is plenty of hard drive space for many weeks worth of data. Thus instead of data from only three nights at a sleep center, a user could get a diagnosis from her last month of sleeping habits. This long-term solution is also viable because the technology is noninvasive and would be practical for a patient to use every night for a month.

Additionally, the system itself can give recommendations and customized reports based on the data it collects. This is discussed in further detail in architecture—software design Section 5.2.

## 5. ARCHITECTURE

### 5.1. Hardware

The hardware we have chosen for PATHOS includes the following.

- Tmote Sky [5]: every sensor node consists of a Tmote Sky with either embedded sensors or sensors attached through analog output. The motes provide the Zigbee wireless connection and are battery powered, allowing complete freedom of placement.
- BCI Micro Power Oximeter Board [6]: an extension for the Tmote Sky that allows a finger pulse oximeter to be connected directly to the mote. This sensor checks blood oxygen level and also records pulse.
- EasySen SBT80—Multi Modality sensor board [7]: another extension board for the Tmote Sky, this board provides additional sensors to the mote, the most important of which are two dual-axis accelerometers which, when combined, can measure motion in three dimensions.
- Handheld device or personal computer: forming the base of the network and is responsible for managing the collected data. The program will be implemented so that it is deployable on the user’s existing hardware, cutting down costs and the amount of devices present.
- Cellular phone: used for collecting data during the day. The daytime collection software can be implemented as either a program that runs on the cell phone or a program running server-side that collects information through text messages or an automated phone system.

### 5.2. Software design

**Figure 2:**
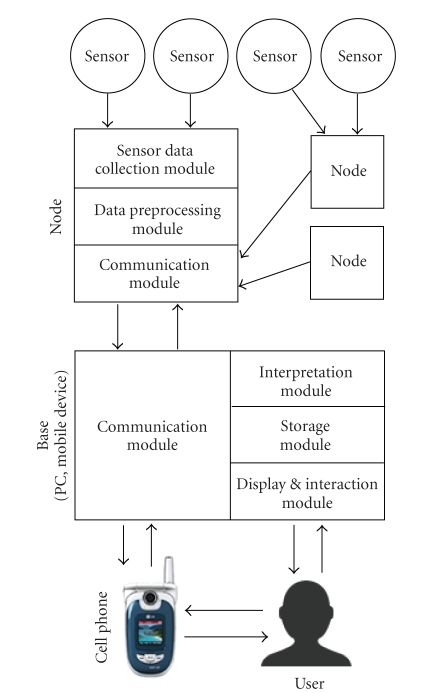
Diagram of the software architecture.

Our software design consists of two separate programs: one running on the nodes and one running on the base station. Figure 2 provides an overview of the programs and how they interact with each other and the user.

The program running on the motes is responsible for collecting data from the sensors and processing the data before sending it to the base. The sensors are either built into the nodes (temperature, humidity, movement), or are provided via a wired external sensor (pulse oximeter, thermistors, acoustic). The nodes run the TinyOS operating system and as a result are quite capable of preprocessing the data and transforming the analog voltage input to a format that can be interpreted easily by the base. As soon as the data is processed, it is sent wirelessly to the base. Nodes also have the ability to relay signals from another node that is too far away to connect to the base. Because the Tmote Sky’s ZigBee wireless has a range of over 50 meters (indoor), a node will typically never be out of reach of the base, unless the wireless is scaled back in order to ensure a more secure connection.

The software running on the base is more important from the user’s point of view because it is the only aspect of the system that he interacts with regularly. The most important part of the base software is the communication module, which is responsible for gathering the data from multiple incoming motes, receiving data throughout the day from a cell phone or internet source, and sending collected data to the doctor for review. Because very few computers or handheld devices implement the ZigBee wireless protocol, a node must be plugged into the handheld device or computer via a USB port. That node then relays all of the information from the incoming nodes by UART to the base.

The interpretation module is responsible for identifying outlying data, for example if a sensor falls off during the night, or becomes disconnected the data will not be included in calculations, but it will still be stored for future reference. The interpretation module is also important because it computes and stores data results as it is collected. For instance, every day the module will calculate averages, maxes, and minimums, and store this data so that it does not have to be calculated again when the user wants to run longer, more detailed reports.

A separate storage module is necessary because it must decide where exactly to store the collected information. Because the system is collecting data all night for up to many months at a time, the amount of space needed can easily exceed the memory on a small handheld device. Normal computer should have little problem storing the data, however in both cases the storage module will utilize data compression techniques to remove statistical redundancy and efficiently store data in the smallest possible space.

**Figure 3:**
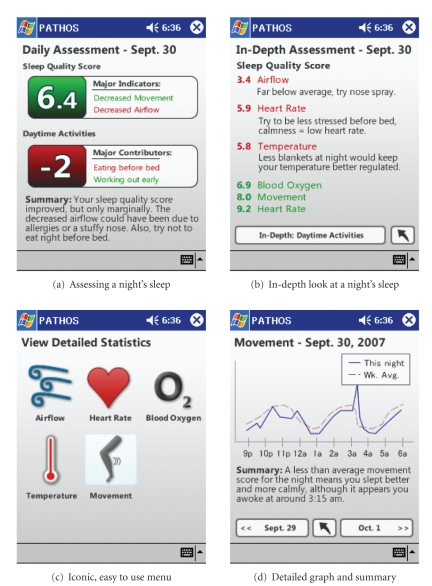
Some screenshots of our implementation.

The display and interaction module is visible in the screenshots in Figure 3. Every morning when a user awakes she has the option to look at a daily assessment of the last night’s sleep. Figure 3(a) shows this function, where the program assigns a sleep quality score based on the collected data. Screenshot 3(a) also shows how the daytime activities affected sleep and a summary of the entire page. The results of the screenshot are simulated to develop such a score would require extensive processing of the data which we have not developed yet. However, it is possible for a doctor to receive a week’s worth of data and then send the patient the information listed in screenshots 3(a) and 3(b). The doctor would make the observations using his own judgment, and when the data is sent to the base it informs the user that a new assessment has been delivered. This would effectively allow a doctor to make a remote diagnosis, and if the condition is severe enough the diagnosis could be done in person as well.

Screenshots 3(c) and 3(d) are fully implemented, as they rely on collected data. The summary section in 3(d) is implemented using a predefined set of threshold values that take into account the users long-term and short-term average scores. Screenshot 3(c) displays the program’s simple, iconic, point-and-click interface (or one style tap, in this case).

Also, in screenshot A the large numbers allow a user to quickly identify how well they slept according to the sensors and what factors during the day influenced their sleep at night. The numbers themselves are clickable, leading to the in-depth assessment found in screenshot 3(b).

## 6. IMPLEMENTATION

**Figure 4:**
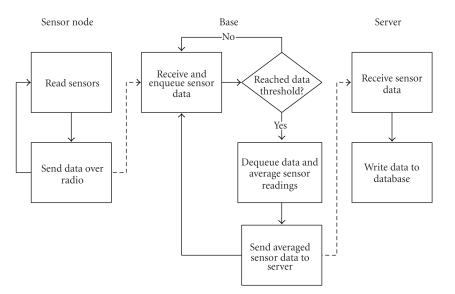
Flow diagram of the implementation.

We have partially implemented the aforementioned hardware and software architecture of PATHOS. The flow of data can be seen in Figure 4. The sensor node collects data and sends it over the radio; the base receives and processes the data for sending to the server, which in turn stores it in a database.

All of the sensors we have implemented are accessed via the Tmotes external ADC ports. They return a voltage that can be converted into the specified units for the type of data the sensor collects. This conversion is performed in the base station in order to keep the mote software simple, so that the formula’s can be modified as necessary.

The base implements a queue for the sensor data it receives in order to process them efficiently and without worry of losing data. After the threshold limit for the queue has been reached, the base program dequeues the data, averages each reading, and sends it to the server. Since the sensor nodes send in data twice a second, an optimal threshold limit is 120 data packets, or one minute’s worth of data. One minute is enough time to average out any sensor hiccups and also a small enough time span in which graphs and analysis can have fine grain data control. Larger limits can be used as well, and we will have to do more research to find the optimal limits for different sensor types.

The server side programming is currently implemented with a PHP file. The base posts the sensor data to the website, and the server then stores the data in a flat file xml system. When the site is accessed by the user in a browser, the sensor data collected over the last hour is displayed. The completed system will have authentication by the base and user account for each individual user. They will be able to login to the website and view statistics for the last day, month, or even year. The website will be the user’s primary means for accessing analysis of the data and doctor’s recommendations, and the data will also be sent to the base for easier display.

## 7. EVALUATION

**Figure 5:**
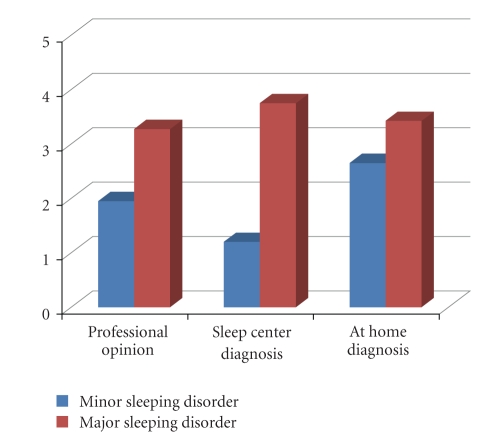
Graph of user response.

To help us evaluate the idea of a home-based sleep monitoring system and continuous information gathering, we took a survey of 20 participants. Our survey confirmed the statement that a home-based system is a better alternative for many people than an overnight sleep center. Figure 5 details the results of our survey, where the subjects were asked if how likely they were to seek professional opinion, if they suspected they had a minor or major sleeping disorder, and then how likely they would go to a sleep center or stay at home for a diagnosis. The results show that the participants were 10% more likely to receive home monitoring than go to a sleep center if they suspected they had a major sleeping disorder. The difference increased dramatically when a minor sleeping disorder was concerned: home monitoring was favored 29% more than a sleep center. Our survey also showed that the participants were twice as likely to seek professional opinion if they suspected they had a major sleeping disorder rather than a minor sleeping disorder.

**Figure 6:**
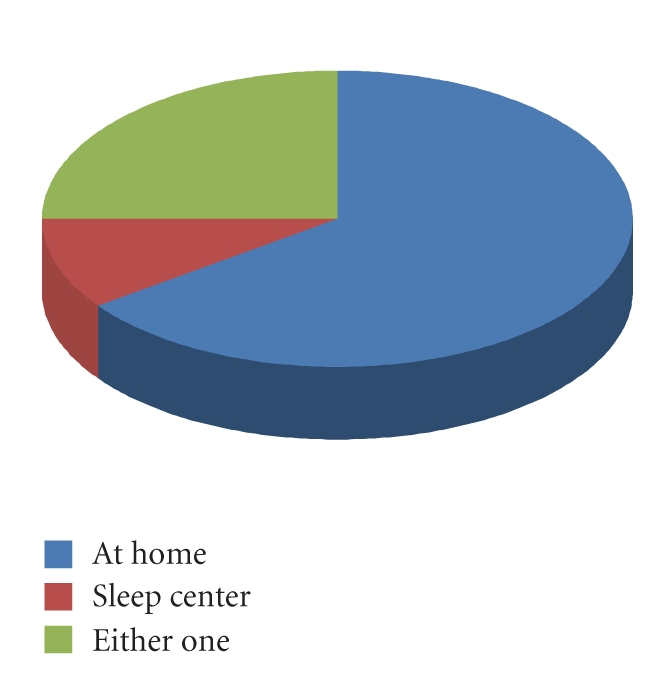
Chart of preference for home monitoring or sleep center.

Finally, in a question where participants were asked outright which they would prefer, only 10% chose the sleeping center, versus 75% who would prefer to have the monitoring done at home. Figure 6 shows the distribution of participants who would prefer at home diagnosis or a sleep center. The majority of the participants would prefer to be monitored at home, which could be due to several reasons, including increased comfort and convenience. This survey data confirms that there is a large need for inexpensive, easy to use home sleep monitoring.

Also, over 70% of the participants said they would be willing to enter data throughout the course of the day if it were convenient and helped the diagnosis of a sleep disorder. Although this figure is favorable, it could be higher, which means a prototype system must be very convenient and easy to use. Another possibility is that the user is asked a short series of questions once or twice per day, which would cut down on the number of times a user has to remember to interact with the system.

**Figure 7:**
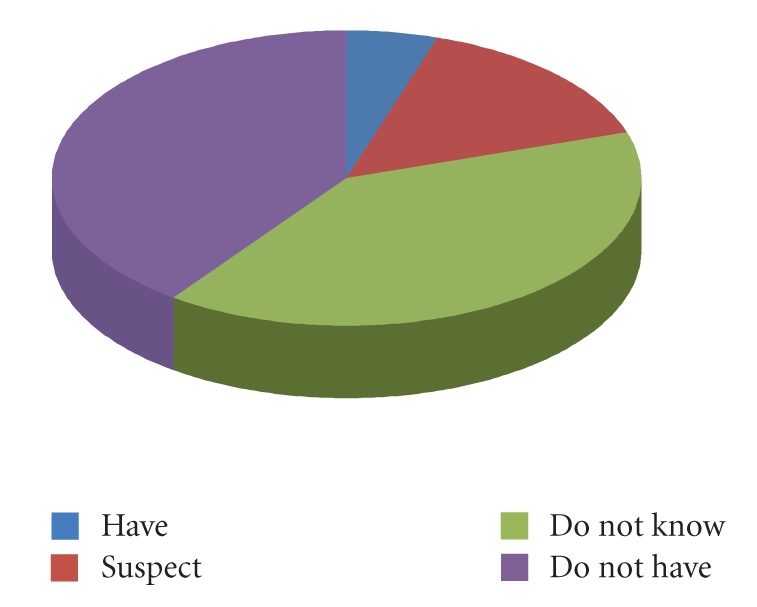
Chart of the participant’s knowledge of their own conditions.

Finally, the participants were asked whether they have, suspect they have, are unsure, or do indeed have a sleeping disorder, the results of which are shown in Figure 7. Even from this small sample of participants, it is clear that there is a need for a simple, at-home sleep monitoring solution because around 40% of participants were unsure whether or not they have a sleeping disorder. If disorders were easier to diagnose, that number would be much lower.

## 8. RELATED WORKS

Many projects are underway that focus on general health monitoring. A long term monitoring system known as Terva [8] has been implemented to collect critical health data such as blood pressure, temperature, sleeping conditions, and weight. The problem with Terva is that although it is self contained, it is housed in a casing about the size of a suitcase, which seriously dampers mobility. As a result, Terva is only practical inside the home. IST VIVAGO is a system used to remotely monitor activity and generate alarms based on received data [9]. In contrast with Terva, our system is small and completely wireless, allowing it to easily adapt to new situations.

Another system, wireless wellness monitor (WWM), is built specifically to manage obesity [10]. The system has measuring devices, mobile terminals (handheld devices), and a base station home server with a database. It uses Bluetooth and Jini network technology and everything is connected through the internet. The MobiHealth project [11] is similar to WWM as it monitors a person’s health data using small medical sensors which transmit the data via a powerful and inexpensive wireless system. A combination of these sensors creates a body area network (BAN), and the project utilizes cell phone networks to transmit a signal on the fly from anywhere the network reaches.

Students at Duke University [5], as part of their DELTA Smart House design, described a system for monitoring sleeping patterns that is easy to use and inexpensive. In order to gather detailed sleep data, they used a pulse oximeter to record the user’s heart rate and respiratory rate, a watch style actigraph to measure movement, in-bed thermistors for body temperature, and a microphone for audio. Their system achieves a low cost by using multifunctional sensors, but their choice of an actigraph adds a considerable amount to cost. Their approach depends on a computer for data interpretation, and the sensors themselves are not actually integrated. For instance, the watch actigraph must be plugged into a computer to transfer data; collection is not seamless.

As part of the SENSATION Project [12] researchers have put together a system using the latest technology to detect sleep and sleepiness. They proposed using a ring that detects heart rate and wirelessly transmits the data, pressure sensitive film to measure chest and limb movement, a microcamera to make sure a driver’s eyes are on the road, and (BAN) technology to have all the parts communicate wirelessly. As apparent by the choice of sensors, the consortium is more focused on preventing driver from falling asleep at the wheel than collecting data for diagnosis of sleeping disorders.

Taking a different, completely noninvasive approach to sleep monitoring, researchers at the University of Tokyo [13] have used the “surrounding sensor approach.” Instead of placing sensors on the subject’s body, they are using motion sensors, cameras, and microphones placed in the surrounding environment to provide noninvasive monitoring. The downside to this approach, although it is meant for home use, is that it is not very portable and therefore must be semipermanent.

Another approach to inexpensive sleep monitoring has been implemented by the University of Washington Seattle with the use of multimodal sensors. As opposed to the expensive actigraph, they investigated the possibility of using a passive infrared camera to record motion during sleep, a decision which carries the same consequences as the surrounding sensor approach, and may be more difficult to setup than sensors that simply attach to the body.

The last system we will review is the FPGA-based sleep apnea screening device for home monitoring developed by researchers at the University of Cairo. The purpose of their system is to determine whether or not a patient should undergo a full polysomnography exam, instead of being used in place of a sleep center. Also, differing from our system, the data is recorded on a Secure Digital card to be processed later by the doctor.

## 9. CONCLUSION AND FUTURE RESEARCH

In this paper, we have presented the details of PATHOS, a hardware- and software-based implementation for monitoring sleeping conditions and lifestyle habits related to sleeping conditions from the comfort of the user’s home. It has been designed to break the field of polysomnography away from the sleep center and bring it into the patient’s home by using wireless connectivity and existing hardware. By doing so, we hope that a more inviting system will lead to the diagnosis of more sleeping disorders and increase the comfort of many people.

In the future, we will be finishing the implementation in order to test the system with a real patient, collecting data and displaying output on a handheld device. We will also be running more extensive test of the graphical user interface. These analyses will allow us to assess the strengths and weaknesses of our design and modify it accordingly. Additionally, we would like to produce algorithms for calculating sleep quality scores and look at how perceived sleep quality matches up with actual sleep quality.
